# Patient-related reasons for late surgery cancellations in a plastic and reconstructive surgery department

**DOI:** 10.1016/j.jpra.2018.08.002

**Published:** 2018-09-05

**Authors:** Liisa Hänninen-Khoda, Virve Koljonen, Tuija Ylä-Kotola

**Affiliations:** Department of Plastic Surgery, University of Helsinki and Helsinki University Hospital, Helsinki, Finland

**Keywords:** Elective operation, Adult, Child, Plastic surgery, Operating theatre efficiency, Communication

## Abstract

Late cancellations of scheduled operations cause direct and indirect costs for a hospital and economic and emotional stress for the patient. Previously, late cancellation rates for scheduled operations in plastic surgery have been shown to be attributable to patient-related causes in the majority of cases.

In this retrospective study, we sought to examine specifically the patient-related reasons for the late cancellations in a plastic surgery operating theatre at Helsinki University Hospital in Finland from 2013 to 2014. We calculated latency between the date of decision for surgery and the scheduled operation day. In cases where the surgery was rescheduled and performed before 31 December 2015, the rescheduled waiting time latency was calculated. We aimed to improve our knowledge of the causes of late cancellations to further optimise the operating theatre efficiency and propose a strategic algorithm to avoid late cancellations

During the study period, 327 (5.5%) of all the scheduled operations were recorded as late cancellations. Of these, 45.3% were because of patient-related issues. Acute infection, change in medical condition not noticed before and operation no longer necessary were by far the most common causes of cancellation, comprising 63.5%. Sixty-six per cent of patient-related cancelled operations were performed later, especially when the specific reason was patient's acute illness. Root-cause analysis shows that most of the underlying reasons for the cancellations can be attributed to a failure in communication. The majority of these cancellations were considered to be preventable, thus emphasising the importance of communication and skilful multi-professional planning of the operating theatre list.

## Introduction

Cancellations of scheduled operations cause direct and indirect costs for a hospital 1, 2, 3 and economic and emotional stress for the patient.^4^ They are a good indicator of hospital and operating room efficiency.^5^, ^6^, ^7^ The overall cancellation rates for scheduled operations vary considerably, ranging from 5% to 40%.^8^, ^9^, ^10^, ^11^ The large range may be explained by differences in how the timing of cancellation is defined in different studies, for example whether the preceding weekday or day of surgery is used as the cut-off point.

Reasons for cancellations vary between countries and even hospitals, depending on several factors such as the characteristics of the population served by the hospital ^12^^,^^13^ or different policies followed by pre-assessment clinics.^14^, ^15^, ^16^ Nevertheless, reasons can be grouped and thus simplified into broader categories such as patient, hospital and staff related.^1^^,^^5^ Cancellation on the day of the surgery, or operations cancelled or rescheduled after 12 p.m. on the preceding weekday are recognised as late cancellations. However, a lack of consensus on the use of the term ‘late cancellation’ may be a reason for the large variation in late cancellation rates, which ranges from 4% to 21%.^1^^,^^5^^,^^9^^,^^17^^,^^18^

The average cancellation rates in plastic surgery are among the cancellation rates of other specialties ^2^^,^^17^, ranging from 4% to 14%.^1^^,^^3^^,^^17^^,^^18^ However, it seems that plastic surgery has a higher proportion of cancellations because of patient-related factors compared with other surgical specialties.^1^^,^^2^ Only two of the previous studies (both from the USA) focus particularly on plastic surgery cancellations. In the study by Guyuron and Zarandy, the overall cancellation rate was 12%, and all the cases were cancelled because of patient-related issues.^18^ In a more recent study focusing on plastic surgery service by Coady-Fariborzian et al., the cancellation rate was 8%, and the majority of reasons for cancellation (65%) were patient related.^3^

Previous literature shows that patient-related cancellations are regular in plastic surgery operations. Therefore, the aim of this study was to examine specifically the patient-related reasons for the late cancellations in a plastic surgery operating theatre at Helsinki University Hospital in Finland from 2013 to 2014. We sought to improve our knowledge of the causes to further optimise operating theatre efficiency and propose a strategic algorithm on the basis of our results.

## Patients and methods

The hospital Institutional Review Board approved this retrospective chart review study and its protocol.

Cancellations of scheduled operations were identified by the electronic OPERA® Operating Room Management System, Töölö Hospital, Helsinki, Finland from 1 January 2013 to 31 December 2014. Patients’ age, gender, type of operation (elective or emergency) and month of cancellation were identified for all late cancelled operations.

During the study period, altogether 5927 operations were scheduled in the operating theatre of the Department of Plastic Surgery, Töölö Hospital, Helsinki, Finland. The majority of patients were females, i.e. 3083 (52%), and males comprised 2844 (48%) of the cases. Of all scheduled operations, 1195 (20.2%) were for paediatric patients and 1508 (25.4%) for patients over 65 years of age. Most of the operations, i.e. 4911 (82.9%), were elective.

Specific inclusion criteria for this study were that the operation was cancelled after 12 o'clock on the preceding weekday, or on the day of the operation, and that the cancellation was recorded using the standardised cancellation code in the electronic OPERA® Operating Room Management System. We included the following standardised patient-related codes in OPERA®:

A01: Patient unfit for operation
A02: Patient unfit for anaesthesia
A03: Patient's acute infection
A04: Patient failed to attend hospital
A05: Patient refused surgery
A06: Need of care changed (no operation)
A09: Patient death
A12: Other patient-related reasons.

Computerised medical records of the patients fitting the inclusion criteria were reviewed in detail for demographic, clinical and treatment characteristics by the first author (LH-K). We collected data on patient age, sex, diagnosis, scheduled operation, the date of decision for surgery, the cancellation date, the date of the rescheduled operation and the specific reason for the cancellation. The cancellation rate was calculated by reviewing all programmed operations (5927), of which 5600 were performed and 327 were cancelled late.

The specific reason for cancellation was organised into seven categories according to the cause found in computerised medical records. The categories were patient's acute illness, change in the patient's medical condition, no need for operation, other patient-related reasons, the cancellation reason could not be found in the records, operation performed already and patient death. Agreement between standardised patient-related codes in OPERA® and the reason for cancellation extracted from the medical records was evaluated. The specific reasons for cancellations were further divided into preventable, partially preventable and non-preventable. In this study, preventable cancellations were considered to be a change in the patient's medical condition not noticed before; no need for operation and operation had been performed already. Patient's acute illness was considered to be partially preventable. Patient death was considered non-preventable.

We determined the latency between the date of decision for surgery and the scheduled operation day: the *scheduled waiting time*. In cases where the surgery was rescheduled and performed from 1 January 2013 to 31 December 2015, the *rescheduled waiting time* latency was calculated.

## Results

### All late cancellations

We recorded a total of 327 late cancellations during the study period. Of these, 148 (45.3%) were because of patient-related issues and 179 (54.7%) because of staff- or hospital-related reasons. Of all the late cancellations, male patients constituted 174 (53%) of the cancelled cases, with a cancellation rate of 6.5%. There were 153 (47%) cancellations for female patients, with a cancellation rate of 5%. Of all the late cancellations, 53 (16.1%) were paediatric patients and 86 (26.1%) were patients over 65 years of age. The highest late cancellation rate was in June: 32 of 430 scheduled operations (7.4%), and the lowest rate was in December: 17 of 438 (3.9%).

### Late cancellations because of patient-related issues

**Figure 1:**
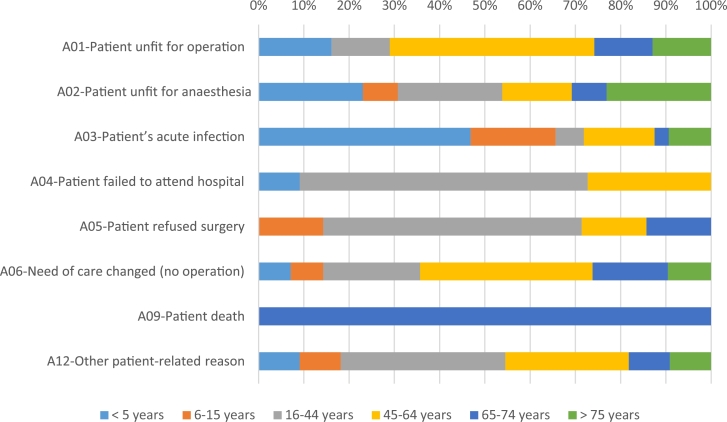
Reasons for cancellation stratified by age groups.

There were 148 cases of late cancellations because of patient-related issues. Our study cohort comprised 85 (57.4%) male and 63 (42.6%) female patients. Most of the patient-related cancellations were recorded in the 45–65 years age group, with 44 (29.7%) patients. Figure 1 presents the late cancellations because of patient-related issues stratified by age group. Most of the late cancellations because of patient-related issues were recorded in August: 18 of 22 (81.8%), and the least number of cancellations was recorded in May: 11 of 37 (29.7%).

The majority were wound- or ulcer-related operations: 33 (22%), followed by cleft lip and palate operations: 22 (15%). The third most common operation type was the excision of cutaneous or subcutaneous lesion in 17 (11%) patients followed by 14 (10%) burn excisions and 13 (9%) corrective procedures.

The specific reasons for cancellations were extracted from the medical records, Table 1. The most common reason for patient-related late cancellation was patient's acute illness in 37 (25.0%) cases followed by change in the patient's medical condition in 29 (19.6%) cases. In 11 (7.4%) of the cases, the scheduled operation had already been done, most commonly on the ward as a bedside procedure.

**Table 1:** Reasons for late cancellations because of patient-related causes.

Reason for cancellation and rescheduled operations	N	Proportion of all patient-related late cancellations %	Proportion of all late cancellations %	Number of rescheduled operations
**Acute illness**	**37**	**25**	**11.3**	**34**
Respiratory infection	27			
**Change in patient medical condition not noticed before**	**29**	**19.6**	**8.9**	**20**
Chronic medical problems	15			
Laboratory values not correct	7			
**No need for operation**	**28**	**18.9**	**8.6**	**7**
Defect healing well or already healed	21			
**Other patient-related reasons**	**25**	**16.9**	**7.6**	**9**
No-show	14			
Patient refused surgery	7			
Timetable problem	3			
**Reason not known**	**17**	**11.5**	**5.2**	**16**
**Operation already performed**	**11**	**7.4**	**3.4**	**11**
Bed-side operation	6			
**Patient death**	**1**	**0.7**	**0.3**	**0**
**Total**	**148**	**100**	**45.3**	**97**

### Cancellation method or place

The preoperative appointment took place either at the outpatient clinic or on the ward either on the day before or on the same day of the scheduled operation. Sixty-four (43.2%) operations were cancelled at the outpatient clinic.

Thirty-eight (26%) in-hospital operations were cancelled on the ward. The most important cause (16, 42.1%) was a change in medical status not noticed before. In addition to these, 13 (8.8%) cases were cancelled after a patient's telephone call to the hospital, and patient's no-show without notification occurred in 13 (8.8%) cases. The cancellation place or method was not known in 19 (12.8%) cases.

### Rescheduled operations

During the follow-up period, 97 (66%) of the patient-related cancelled operations were performed eventually. The operation took place especially if the specific reason for cancellation was patient's acute illness: 92% (34/37) were rescheduled and operated. Sixty-nine per cent (20/29) of cancellations were performed later in the ‘change in patient's medical condition’ group. If the reason was no need for operation, the operation was performed in 25% (7/25) of cases. In five cases, the rescheduled operation was cancelled one more time because of patient-related causes.

Almost half of the cancelled operations at the outpatient clinic were performed later: 17 (49%). Of the ward-cancelled operations, 22 (76%) were rescheduled and performed later. In the case of in-hospital patients, 19 of the cancelled cases (50%) were operated later in the follow-up period.

### Scheduled waiting time

The mean scheduled waiting time was 94 days, range 1–473 days, and for 33.8%, it was from 1–6 months. In Table 2, scheduled waiting times according to the cancellation reasons are illustrated. In 12 (8.1%) cases, the scheduled waiting time could not be defined because of missing information.

**Table 2:** Scheduled and re-scheduled waiting times stratified by reason for cancellation.

	Up to < 2 days		2-7 days		1-4 weeks		1-6 months		6-12 months		Over 1 year	
	Scheduled waiting	Re-scheduled waiting	Scheduled waiting	Re-scheduled waiting	Scheduled waiting	Re-scheduled waiting	Scheduled waiting	Re-scheduled waiting	Scheduled waiting time	Re-scheduled waiting time	Scheduled waiting time	Re-scheduled waiting time
**Change in medical condition**	5	3	7	7	5	3	5	4	3	2	2	1
**Patient's acute illness**	0	0	3	3	7	10	17	20	8	0	1	1
**No need for operation**	5	0	5	0	5	1	8	5	3	0	0	1
**Other patient-related issues**	1	0	4	2	3	2	7	3	10	0	0	2
**Reason not known**	1	2	2	2	3	6	8	5	1	1	0	0
**Operation performed already**	3	11	3	0	0	0	1	0	0	0	0	0

### Rescheduled waiting time

The mean rescheduled waiting time was 84 days, range 0–956 days. It was under 1 week in 2.1%, 1 week to 1 month in 25.6%, 1–6 months in 43.0% and more than 6 months in 9.3% of cases. Table 2 shows rescheduled waiting times according to the cancellation reasons.

### Preventable and non-preventable cancellations

We determined that 130 (99.2%) cancellations could have been totally or at least partially prevented. Preventable cancellations (93, 71.0%) were a change in the patient's medical conditions not noticed before; no need for operation and operation had been performed already. Patient's acute illness, especially respiratory infections (27, 20.6%), was considered to be partially preventable. Patient deaths were considered to be non-preventable. The specific reason was not identifiable in 17 (11.5%) cases, and these were excluded from analyses.

### Agreement between standardised cancellation codes in OPERA® and reasons for cancellations

**Figure 2:**
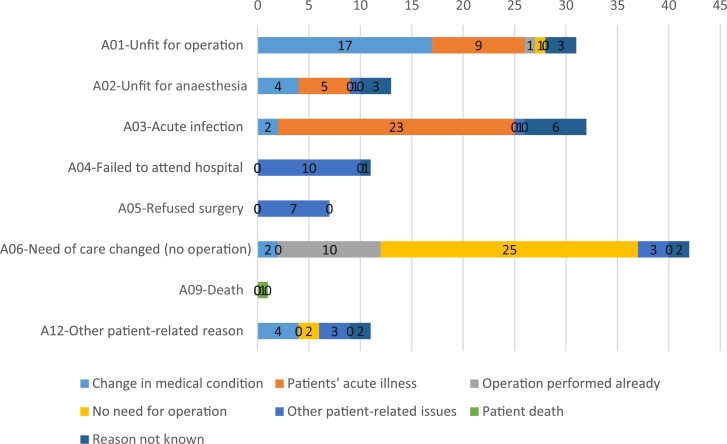
Agreement between standardised patient-related codes in OPERA® and the reason for cancellations extracted from the medical records.

The correlation between the standardised cancellation codes in Opera® and the reason for cancellation found in the computerised medical records of the patients varied greatly. There was agreement in 88 (59.5%) cases. The best correlation was in the group A05 ‘Patient refused surgery’: 7 of 7 cases (100%). Agreement between the standardised patient-related code in OPERA® and the reason for cancellation extracted from the medical records is shown in Figure 2.

### Proposed strategic algorithm

**Figure 3:**
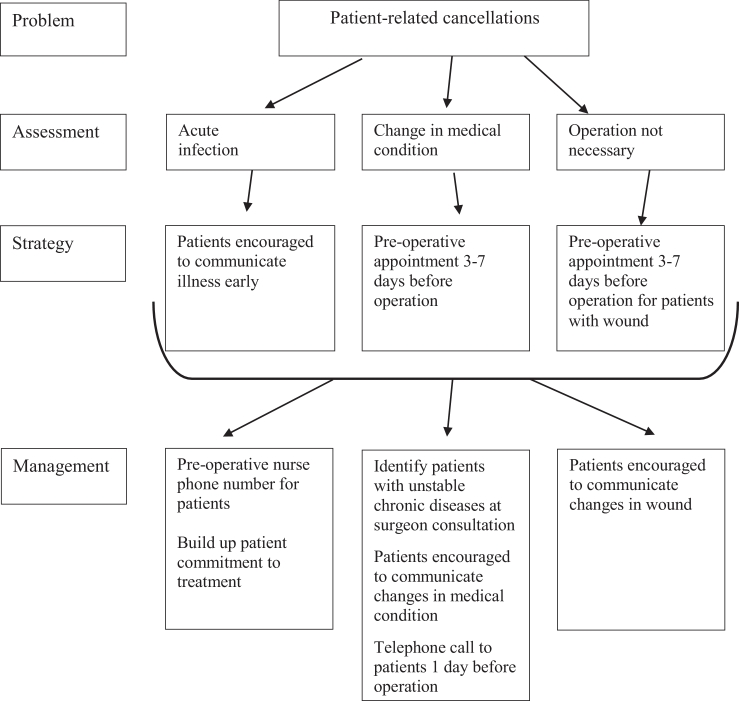
Prosed strategic algorithm for reducing patient-related cancellations.

On the basis of the results of this study, we propose strategic algorithm for reducing patient-related cancellations, Figure 3. The main goal is to encourage patients to communicate changes early, either acute illness, change in their medical condition or their wounds.

## Discussion

We studied the late cancellations because of patient-related issues during a 2-year period in a plastic surgery operating theatre. We recorded 148 late cancellations because of patient-related issues that comprised 45% of all our late cancellations, which is in line with the previous literature.^1^^,^^2^^,^^19^ We estimated that our crude late cancellation rate was approximately 5.5%; however, due to the retrospective nature of our study, it is possible that not all late cancellations were captured in this study as some might have been misrecorded. Despite being in settings not exactly the same as ours, same day cancellation rates in plastic surgery ranged between 4% and 14% ^1^^,^^17^^,^^20^; hence, our late cancellation rate fell well within these numbers. Working-aged patients with wound or ulcer diagnosis represented majority of our patient-related late cancellations.

The main results of our study were that patient's acute infection, change in patient's medical condition not noticed before and operation no longer necessary were by far the most common causes of cancellation, comprising 63.5% of cases. Thus, our findings are in line with those of the previous literature.^1^^,^^2^^,^^5^ Acute illness, especially acute infection, is a common reason for late cancellation among paediatric patients*.*^21^, ^22^, ^23^ Change in medical condition, in this study 20%, is a previously well-established reason for late cancellation, also in plastic surgery.^2^^,^^17^^,^^24^ Identification of patients, who are likely to have change in their medical condition, such as unstable chronic diseases, should be emphasised in the light of our results. This identification should have already been performed at surgeon consultation. Criteria for different pre-operative evaluation are based on the risk of the procedure and whether a patient has chronic disease.^25^ Preoperative evaluation for high-risk surgical procedure and/or unstable chronic disease and/or uncertain functional capacity, requires a multisystem evaluation.^25^ Centralising and standardising routines for pre-operative evaluation may also reduce the cancellations because of changes in medical condition.^26^ In a study from Britain, most of the preventable cancellations could have been addressed at preoperative evaluation, such as following up of abnormal results and comorbidities needing treatment.^27^

We estimated that 99.2% of our late patient-related cancellations could have been totally or at least partially prevented. We consider acute infection partially preventable because even though it might not have been possible to prevent the infection itself, the late cancellation could have been prevented if the operating theatre had been informed early enough. If the cancellations because of acute infection are excluded, we consider that 74.2% of the late cancellations were totally preventable. Our figure is somewhat higher than those reported in previous studies. Schofield et al. estimated that approximately 60% of on-the-day-of-surgery cancellations of elective surgery were potentially avoidable.^20^ Potentially avoidable cancellations do not generally include cancellations because of patient-related reasons or issues. Schofield et al.’s list of avoidable cancellation reasons included mainly hospital, hospital logistics or administrative reasons.^20^

In this current study, 66% of patient-related cancelled operations were performed later, especially when the specific reason was patient's acute illness. Interestingly, when the reason was no need for operation, the operation was still performed later in 25% of cases. Dimitriadis et al. suggested that disagreement with the patient management plan and operation set by another consultant or resident may lead to cancellation or postponement of the operation.^5^ In a Brazilian study, one of the most frequent reasons for same day cancellation was also the request of the surgeon/change of approach.^28^ It is also possible that the condition requiring the operation might have improved or resolved, and the operation is no longer necessary, especially if the waiting lists for operation are long.^5^ This seems to be the case in our study, as 28 cases cancelled in this category were ulcer patients (10 of 28) waiting for either ulcer revision or reconstruction.

Surgical operations are responsible for large costs and health improvements in the healthcare system.^2^^,^^5^ Optimising operating theatre efficiency is therefore vital to improve the healthcare system.^4^ This theme has been studied earlier especially to improve preoperative policlinics.^5^^,^^29^^,^^30^ A hospital with a preoperative clinic has a lower late cancellation rate than that without a preoperative clinic.^2^ Further, studies show a lower late cancellation rate for on-the-day-of-surgery arriving patients than for in-house patients.^12^ All cancelations are not preventable, *e.g*. in cases of acute illness; therefore, it could be advisable to have patients on standby to be added to a next day operation list. In-house patients waiting for non-elective surgery are good candidates for this kind of waiting list.

Previously, Laisi et al. suggested that the interpretation of categorisation instructions may vary between individuals.^1^ In the current study, we were able to compare the standardised cancellation categories with more specific reasons in the clinical files in over 85% of the recorded cases. Our study shows that the best coherence was in the group ‘Patient refused surgery’.

Root-cause analysis showed that most of the underlying reasons for our cancellations can be attributed to a failure in communication. Previously, Singhal et al. reduced the number of patient-initiated cancellations by enhancing communication with the patients closer to the scheduled date of operation. Their simple technique resulted in reducing the rate of cancellation from 10% to 1.6%.^31^ We established three types of communication: patient–doctor, patient–hospital and communication within the hospital. Patient–doctor communication has a pivotal role in the education of, and motivation for, patients to assist in shared decision-making*.*^32^ Good communication between patient and doctor correlates with better outcomes, better adherence to preoperative preparation, better in-hospital care and earlier detection of post-discharge complications.^33^ We should make it easier for the patients to feel comfortable when communicating with the hospital even if they merely suspect but are not sure of, for example, acute infection before the scheduled operation, and in general, we should make it clearer what actions we would like them to take in different situations before the operation. Provision of the relevant information on the surgical procedure seems to be best provided by written information leaflets.^34^

Although the category ‘Operation already performed’ included only 11 patients, resulting in 7.4% of our patient-related late cancellations, it is worth discussing. In this current study, all the operations in this category, Table 2, were performed at our own hospital and within 24h of the scheduled operation, not at a private practice or a different hospital. Clearly, we have a communication breakdown within the hospital. All the data are currently stored electronically, and treatment decisions are stored and distributed in various patient management systems. Although reliable, the management systems may be prone to human errors, such as not removing the patient from the electronic operation waiting list.

Limitations of this study include its retrospective nature. We were not able to determine the reason for 17 (11.5%) of the cancellations because no specific reason was recorded in the medical records. Further, our data were collected from medical records initially not intended for research use. Strengths of this study include using administrative, electronically saved data and combining them with clinical patient records.

To conclude, the late cancellation rate was 5.5%, and 45% of all our late cancellations were due to patient-related factors. The majority of these cancellations were considered to be preventable, thus emphasising the importance of communication and skilful multi-professional planning of the operating theatre list.

## Conflict of interest statement

Each author declares no financial conflicts of interest with regard to the data presented in this manuscript. The funding for this article was from departmental sources only.

## Role of the funding source

The funding for this article was from departmental sources only.
